# Supplementary figures and images for: Temporal and spatial distribution of histone acetylation in mouse molar development
Source: PeerJ. 2025 Mar 31;13:e19215. doi: 10.7717/peerj.19215 (PMC11967410; doi:10.7717/peerj.19215)

● Epi ● IEE ● Mes/DP

A

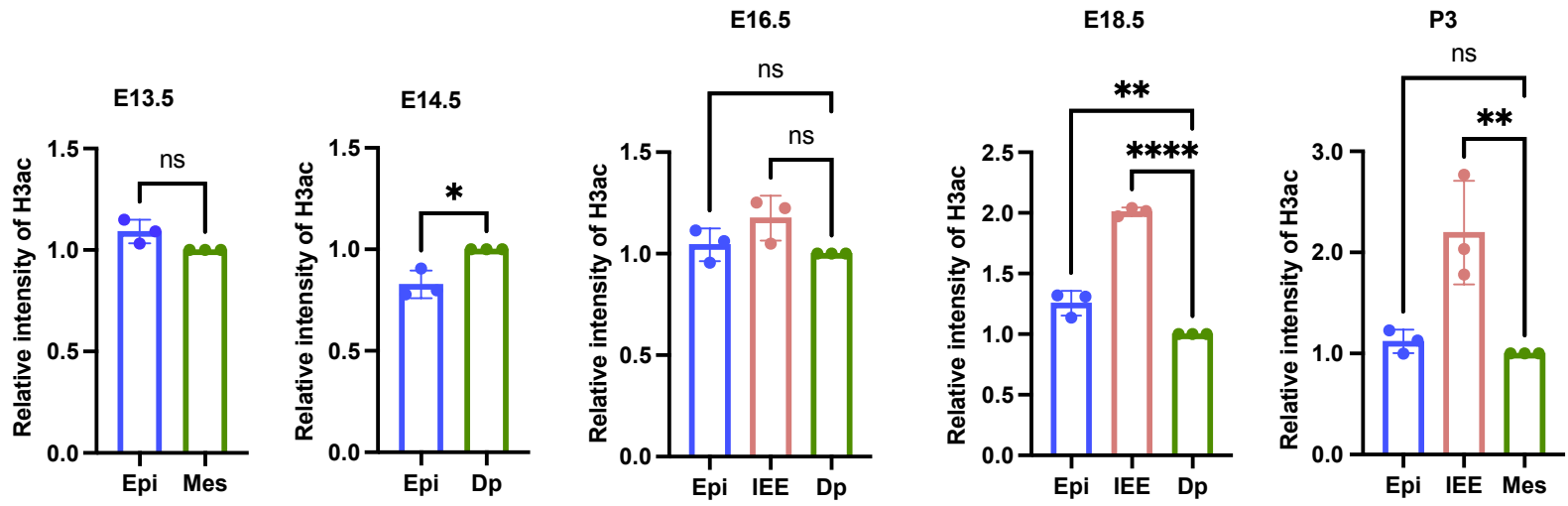

B

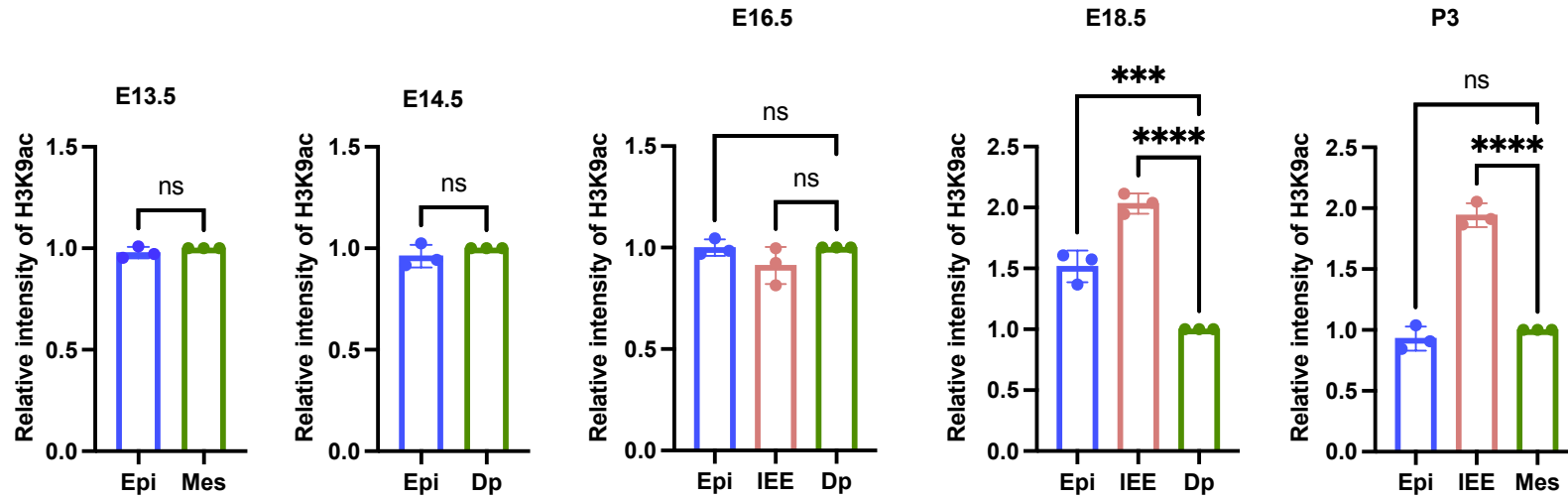

C

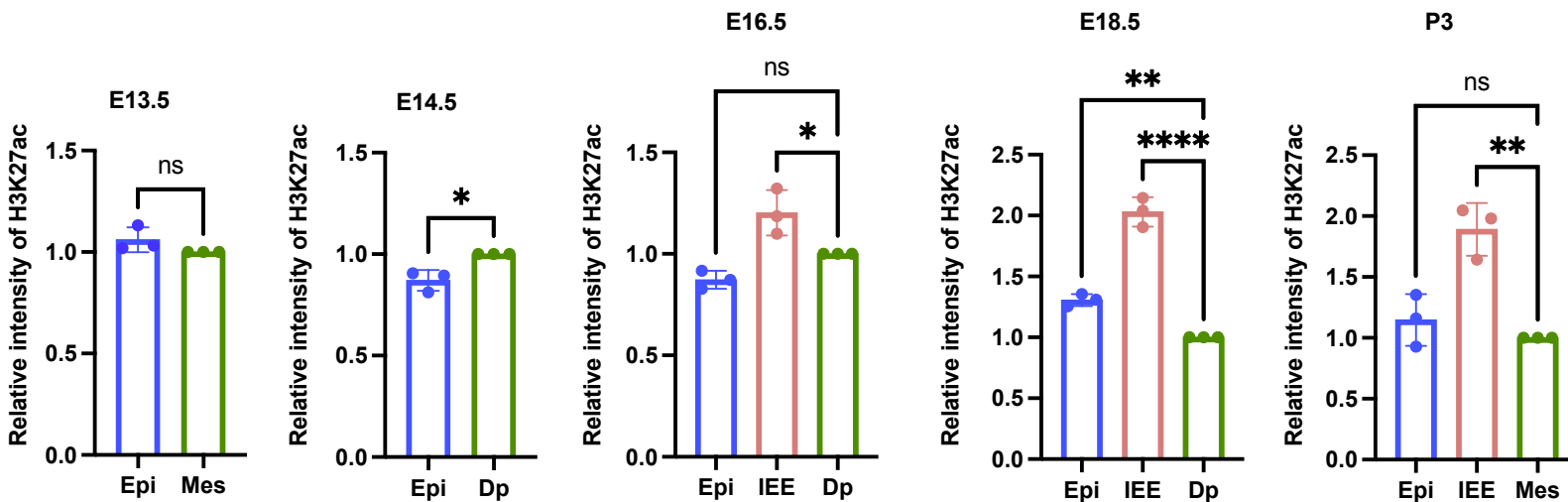

Supplement: Supplemental Information 2 — (A) Quantification of H3ac expression in dental epithelium and mesenchyme/ dental papilla at E13.5, E14.5, E16.5, E18.5 and P3. (B) Quantification of H3K9ac expression in dental epithelium and mesenchyme/ dental papilla at E13.5, E14.5, E16.5, E18.5 and P3. (C) Quantification of H3K27ac expression in dental epithelium and mesenchyme/ dental papilla E13.5, E14.5, E16.5, E18.5 and P3. Epi, epithelium; Mes/DP, mesenchyme/ dental papilla; IEE, inner enamel epithelium. [file peerj-13-19215-s002.pdf]

● Epi    ● IEE    ● Mes/DP

A

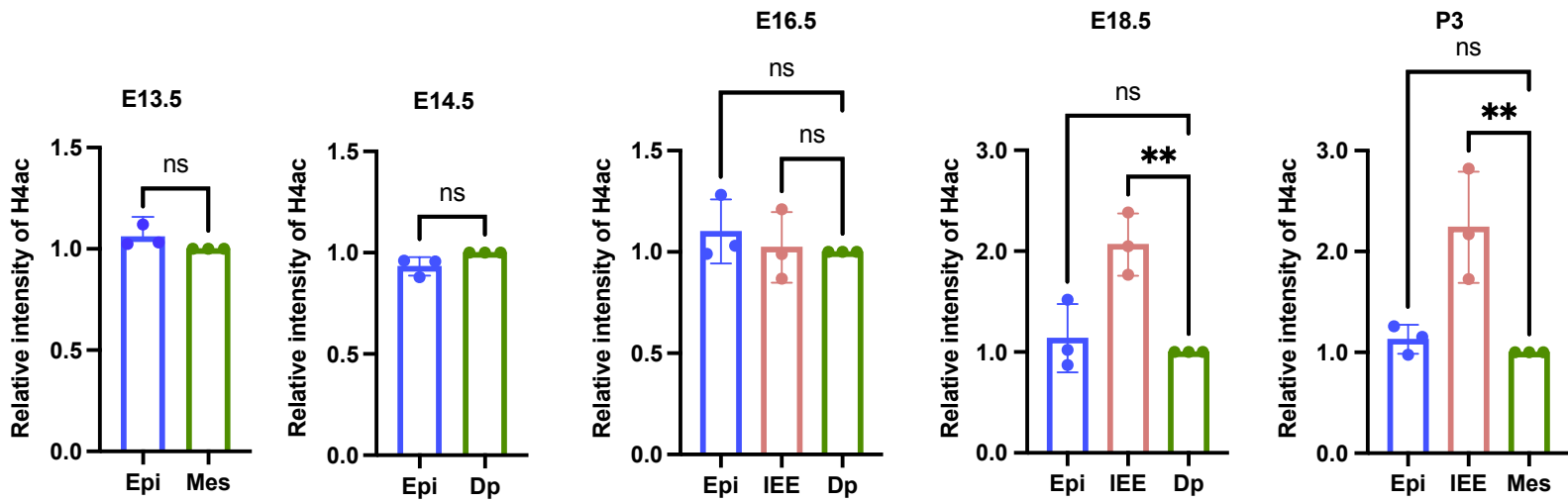

B

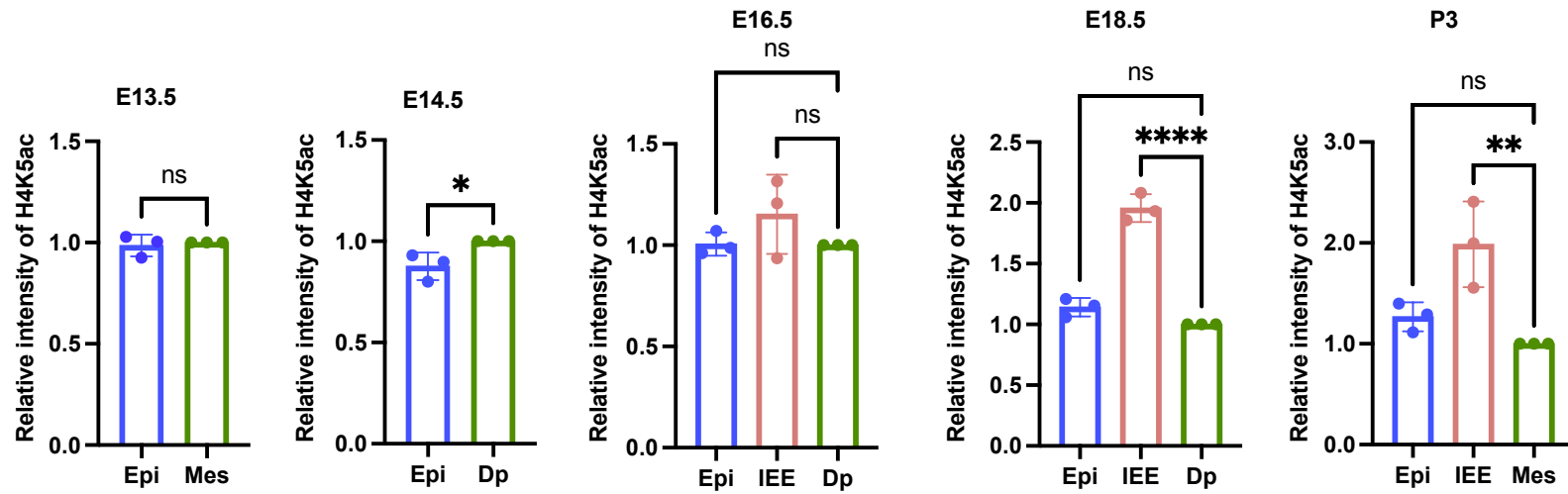

B

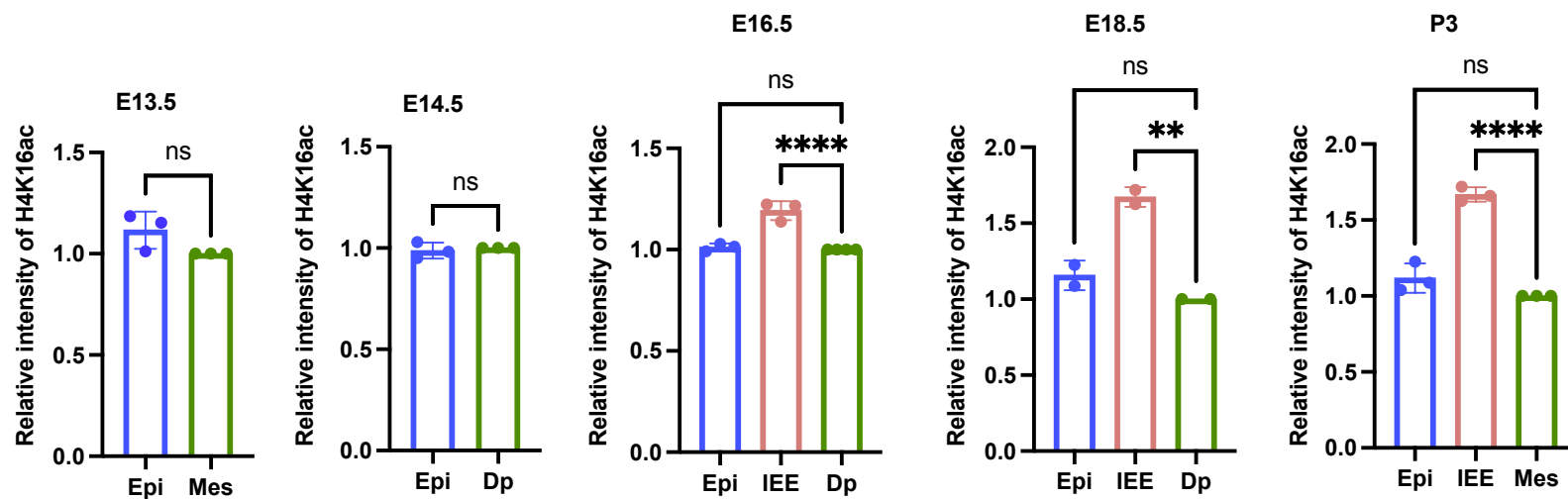

Supplement: Supplemental Information 3 — (A) Quantification of H4ac expression in dental epithelium and mesenchyme/ dental papilla at E13.5, E14.5, E16.5, E18.5 and P3. (B) Quantification of H4K5ac expression in dental epithelium and mesenchyme/ dental papilla at E13.5, E14.5, E16.5, E18.5 and P3. (C) Quantification of H4K16ac expression in dental epithelium and mesenchyme/ dental papilla E13.5, E14.5, E16.5, E18.5 and P3. Epi, epithelium; Mes/DP, mesenchyme/ dental papilla; IEE, inner enamel epithelium. [file peerj-13-19215-s003.pdf]

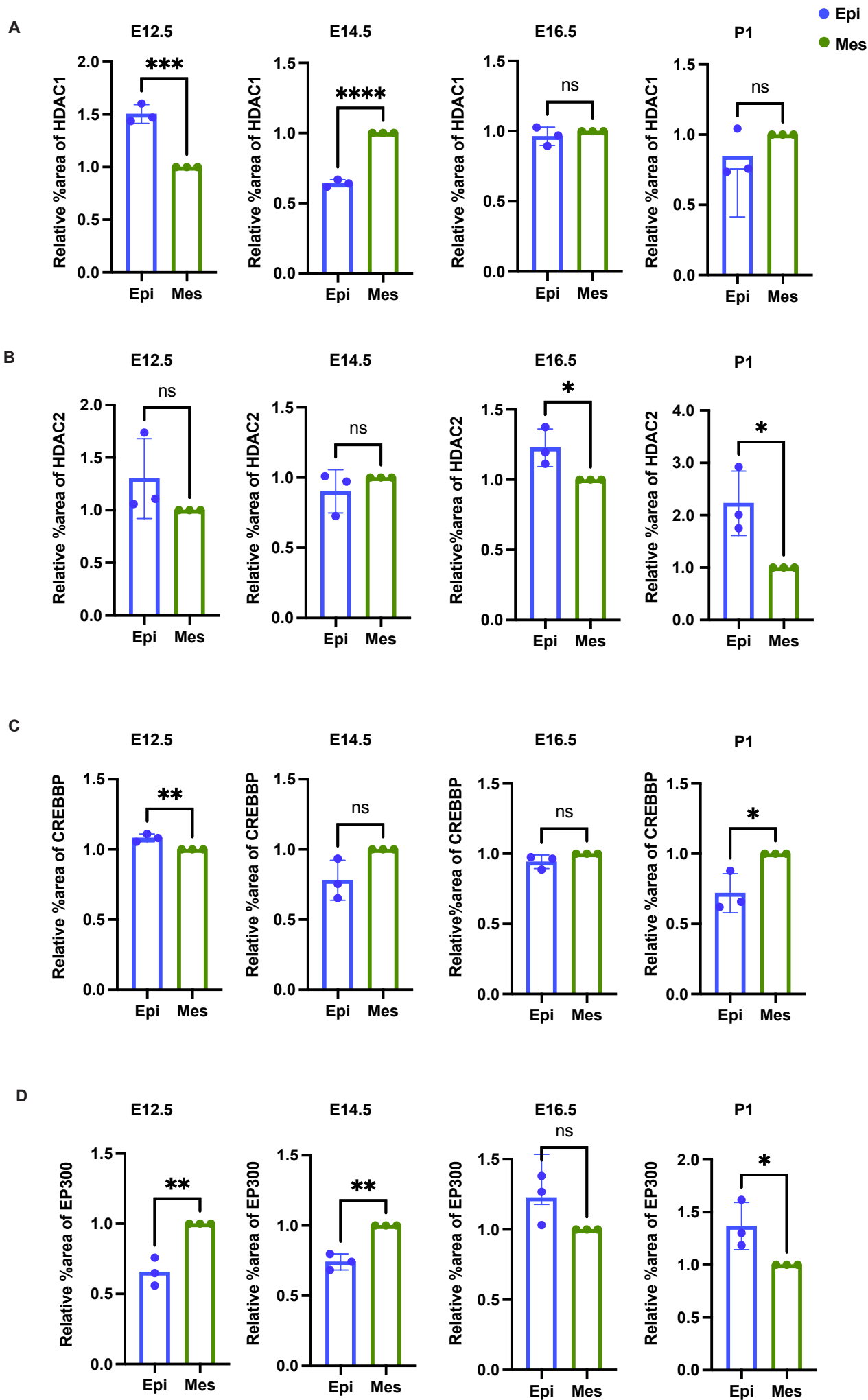

Supplement: Supplemental Information 4 — (A) Quantification of CREBBP expression in dental epithelium and mesenchyme at E12.5, E14.5, E16.5 and P1. (B) Quantification of EP300 expression in dental epithelium and mesenchyme at E12.5, E14.5, E16.5 and P1. (C) Quantification of HDAC1 expression in dental epithelium and mesenchyme at E12.5, E14.5, E16.5 and P1. (D) Quantification of HDAC2 expression in dental epithelium and mesenchyme at E12.5, E14.5, E16.5 and P1. Epi, epithelium; Mes, mesenchyme. [file peerj-13-19215-s004.pdf]
